# Exploring the Link Between Profuse Warts and Hyper-Immunoglobulin E (IgE) Syndrome: A Pediatric Case Report

**DOI:** 10.7759/cureus.61986

**Published:** 2024-06-09

**Authors:** Hassnae Tkak, Madiha Benhachem, Ayad Ghanam, Aziza Elouali, Abdeladim Babakhouya, Maria Rkain

**Affiliations:** 1 Department of Pediatrics, Mohammed VI University Hospital, Faculty of Medicine and Pharmacy, Mohammed First University, Oujda, MAR

**Keywords:** recurrent infections, child, immune deficiency, wart, hyper-ige syndrome

## Abstract

The relationship between warts and hyper-immunoglobulin E (IgE) syndrome lies in the fact that patients with this syndrome may have recurrent or persistent skin warts because of their immune dysfunction. Therefore, it is important to consider this possibility when evaluating a patient with skin warts, especially if they are associated with other symptoms such as recurrent infections or pulmonary issues. Warts can thus be an important clinical sign indicating the presence of this syndrome. We report the case of a young girl presenting with numerous warts accompanied by pulmonary involvement and weight delay, in whom the diagnosis of hyper IgE syndrome was established.

## Introduction

Hyper-immunoglobulin E (IgE) syndromes are rare primary immune disorders in children, involving abnormalities in T lymphocytes, particularly Th17 cells, hyperactivation of B lymphocytes leading to elevated levels of IgE in the blood, impairment of neutrophils, essential immune cells for fighting bacterial infections, and genetic mutations typically affecting the STAT3 gene. It manifests as a triad of symptoms, including atopic dermatitis, recurrent skin and lung infections, and high levels of IgE. These syndromes were initially identified starting from 1966 when a disease characterized by recurrent skin and lung infections, caused by Staphylococcus, alongside eczematous dermatitis, was recognized. This clinical picture was termed Job syndrome [1,2].

The cutaneous findings of hyper-IgE syndrome described in the initial reports and subsequent case series are characterized by an eczematous or atopic dermatitis-like eruption with multiple skin abscesses. Although some descriptions of the cutaneous findings depict an eruption "not typical of atopic eczema," the cutaneous morphology has not been thoroughly or systematically described. Only a few reports have mentioned the initial findings [3].

## Case presentation

This is a two-year-old and nine-month-old girl. She is born to first-degree consanguineous parents. She had two brothers who died in the neonatal period because of an unknown polymalformative syndrome. In her medical history, there were recurrent respiratory infections noted since the age of six months. Otherwise, she had not experienced any skin abscesses previously.

She was admitted for respiratory symptoms characterized by a productive cough evolving two weeks before her admission in the context of a febrile condition and general deterioration marked by a weight loss of 2 kg. A clinical examination revealed a malnourished patient, presenting with weight delay with a weight of 9.5 kg, which is -3 standard deviations, a height of 85 cm, which is -1 standard deviation, and a body mass index of 13.5 kg/m².

**Figure 1:**
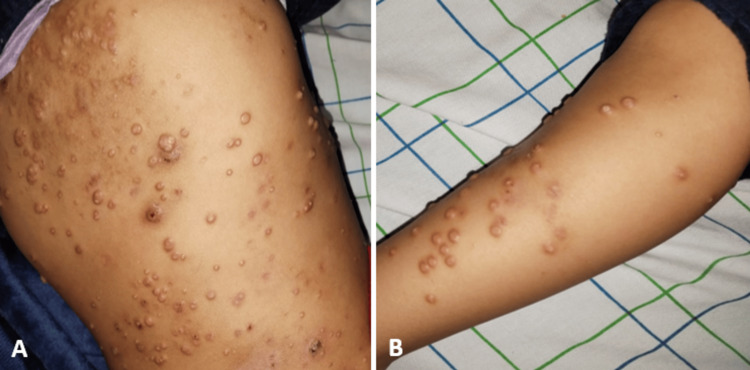
Profound warts on the trunk (image A) and the limbs (image B).

The cutaneomucous examination revealed dry skin and numerous warts scattered throughout the body, suggesting a profuse molluscum contagiosum (Figure 1). Pulmonary auscultation revealed diffuse wheezing and crackles. The rest of the clinical examination was unremarkable.

**Figure 2:**
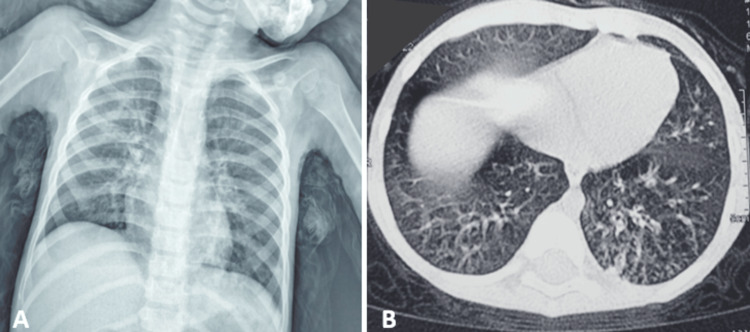
Image A: The frontal chest X-ray reveals a bilateral alveolo-interstitial syndrome with a predominance on the right side. Image B: The thoracic CT scan without contrast injection, in axial sections and parenchymal window, reveals bilateral lung involvement predominantly at the right base.

The frontal chest X-ray showed bilateral alveolo-interstitial syndrome with a predominance on the right side (Figure 2). A complementary thoracic CT scan revealed bilateral lung involvement predominantly at the right base without evidence of bronchial dilatation or pneumatocele (Figure 2).

On a biological level, the blood count revealed lymphopenia and slight eosinophilia, as well as the presence of an inflammatory syndrome with thrombocytosis, elevated sedimentation rate, and C-reactive protein, along with hyperferritinemia. Fibrinogen levels were slightly elevated, and hypergammaglobulinemia was noted. The impact assessment showed hypoalbuminemia along with vitamin D deficiency (Table 1).

**Table 1:** Our patient's biological findings. Hb: hemoglobin; MCV: mean corpuscular volume; MCHC: mean corpuscular hemoglobin concentration; WBC: white blood cell; lymph: lymphocyte; Neut: neutrophil; Eos: eosinophilia; Plt: platelets; CRP: C-reactive protein; ESR: erythrocyte sedimentation rate; PCT: procalcitonin; Alb: albumin; Vit D: vitamin D

Laboratory parameter	Test results	Reference range
Hb (g/dL)	13.3	11-13.5
MCV (fL)	79	80-98
MCHC (%)	27	27-32
WBC (/µ)	7,900	5,200-11,000
Lymph(/µL)	1,950	2,300-5,400
Neut (/µL)	3,500	1,500-7,000
Eos(/µL)	620	0-500
Plt (/µL)	631,000	150,000-400,000
CRP (mg/L)	52	<5
ESR (mm/h)	78	<20
Fibrinogen (g/L)	4.4	2-4
Ferritinemia (ng/mL)	366	15-150
PCT (ng/mL)	0.05	<0.5
Alb (g/L)	27	35-55
Vit D (ng/mL)	7	30-40

Given this array of clinical evidence including growth delay, recurrent respiratory infections, generalized viral skin infection, consanguinity, and lymphopenia, an immune deficiency was considered. A minimal immune deficiency assessment was therefore conducted: human immunodeficiency virus (HIV) serology was negative, and the quantitative measurement of immunoglobulins showed elevated immunoglobulin E (UI/mL) levels exceeding 2,000 UI/mL, with slightly decreased immunoglobulin M levels. Additionally, lymphocyte subset counts were normal (Table 2).

**Table 2:** Our patient's immune deficiency assessment. IgG: immunoglobulin G; IgA: immunoglobulin A; IgM: immunoglobulin M; IgE: immunoglobulin G; NK: natural killer

Laboratory parameter	Test results	Reference range
HIV1/2 (P24 antigen and HIV 1/2 antibodies screening)	0.1	<1
IgG (g/L)	7.2	3.4-6.2
IgA (g/L)	1.9	0.33-1.22
IgM (g/L)	0.45	0.48-1.43
IgE (UI/mL)	2,024	<60
T cell CD3^+^(/mm^3^)	2,029	1,400-3,700
T cell CD4^+^(/mm^3^)	960	700-2,200
T cell CD8^+^(/mm^3^)	605	490-1,300
NK cell CD16^+^/56^+^(/mm^3^)	551	130-720
B cell CD19^+ ^(/mm^3^)	393	390-1,400
B cell CD20^+^ (/mm^3^)	280	200-500

Furthermore, tests for pulmonary tuberculosis and cystic fibrosis were performed, all yielding negative results. Genetic analysis has been performed to identify specific genetic mutations associated with hyper-IgE syndrome, particularly the STAT3 and DOCK8 genes, which are frequently implicated in this disease.

The patient was placed on curative and preventive antibiotic therapy with cotrimoxazole, as well as monthly intravenous immunoglobulin infusion while awaiting a bone marrow transplant. Her clinical condition improved, and she regained weight.

## Discussion

The hyper-IgE syndrome, known as Job's syndrome, is a rare multisystemic disease that manifests from birth or early childhood. It can be inherited through two main modes of genetic transmission: autosomal dominant or autosomal recessive. The genetic mutations involved in this syndrome often affect genes related to the immune system, thereby altering immune response and increasing susceptibility to infections. Its genetic origin was identified in 2007 for the autosomal dominant form and in 2009 for the recessive form [2].

The hyper-IgE syndrome presents a wide range of symptoms. In addition to recurrent skin abscesses, types of pneumonia with pneumatocele formation, and elevated serum IgE levels, this syndrome can also manifest with other symptoms such as dental issues such as misaligned or missing teeth, bone abnormalities such as joint hyperextensibility or spinal curvature, facial anomalies such as a broad forehead, large eyes, and prominent jaw, recurrent upper respiratory tract infections, and various cutaneous complications. These diverse clinical manifestations make the diagnosis and management of this syndrome complex [3].

Initially reported in 1966 by Davis et al. [1], this pioneering study, along with subsequent reports by Buckley et al. [4] in 1972 and Hill et al. [5] in 1974, described "eczematous eruptions" appearing from an early age, followed by the development of skin abscesses. Buckley et al. also documented a "pustular skin eruption" in a six-week-old baby [4]. Although terms such as "eczematous" or "atopic dermatitis" are commonly used in medical literature, the skin eruptions of hyper-IgE syndrome do not fit the diagnostic criteria for atopic dermatitis. Unlike infantile atopic dermatitis, the initial eruption of hyper-IgE syndrome primarily presents as papulopustules on the scalp, face, neck, armpits, and diaper area [3]. Furthermore, this syndrome should be considered in any child presenting with profuse warts, resistant to treatments, or associated with recurrent bacterial infections [2].

The diagnosis of hyper-IgE syndrome relies on a thorough clinical evaluation, meticulous review of medical history, and immunological tests, with elevated serum IgE levels often being key indicators. In cases of suspicion, genetic analysis can be conducted to detect specific mutations, notably in the STAT3 gene (for the dominant form) and DOCK8 gene (for the recessive form) [2,6]. Precise diagnosis often requires the collaboration of a multidisciplinary team including immunologists, dermatologists, and other specialists [2]. Other recessive mutations, such as those in the TYK2 and PGM3 genes, have been associated with hyper-IgE syndrome [7], as have other genetic anomalies leading to hyper IgE, eczema, and dysimmune manifestations. These include Wiskott-Aldrich syndrome, Wiskott-Aldrich 2 syndrome, immune dysregulation, polyendocrinopathy, enteropathy X-linked (IPEX) syndrome, Omenn syndrome, atypical DiGeorge syndrome, and Netherton syndrome. These conditions are very rare and typically manifest in early childhood, with survival to adulthood being exceptionally rare [8,9].

Therapeutic strategies for dominant hyper-IgE syndrome (HIES) primarily focus on treating and preventing skin and lung infections and their complications [10]. Skin or lung abscesses may require prolonged antibiotic treatment and sometimes surgical intervention. Infection prevention involves vaccination and long-term antifungal and antibiotic prophylaxis targeting common pathogens such as staphylococci, pneumococci, and Haemophilus. Intravenous immunoglobulins are effective in reducing bacterial pneumonia. Good skin hygiene is also essential. For recessive hyper-IgE syndrome, characterized by early-onset viral skin infections, lymphopenia, and a more severe course, bone marrow transplantation is the treatment of choice. Antibiotics and intravenous immunoglobulins are used to control infections while awaiting transplantation, and antivirals may be necessary for severe viral infections [7].

## Conclusions

The presence of a high IgE level in a child with profuse warts and recurrent infections may suggest the presence of hyper-IgE syndrome, a rare disease with varied manifestations from early childhood. The clinical case mentioned highlights this relationship, emphasizing the importance of considering hyper-IgE syndrome in the diagnosis of persistent skin warts. The diagnosis of this syndrome relies on thorough clinical evaluation as well as immunological tests, sometimes complemented by genetic analysis. Treatment aims to prevent and treat infections, adjusting approaches based on the form of the disease. Although challenges persist, medical advancements offer promising prospects for enhancing the management of this complex condition.
